# Neuroendocrine Tumor: A Rare, Aggressive Tumor of the Gallbladder

**DOI:** 10.7759/cureus.5571

**Published:** 2019-09-05

**Authors:** Ishtiaq Hussain, Deepika Sarvepalli, Hammad Zafar, Sundas Jehanzeb, Waqas Ullah

**Affiliations:** 1 Gastroenterology, Cleveland Clinic Florida, Weston, USA; 2 Internal Medicine, Guntur Medical College, Guntur, IND; 3 Internal Medicine, AdventHealth Orlando, Orlando, USA; 4 Internal Medicine, Khyber Teaching Hospital, Peshawar, PAK; 5 Internal Medicine, Abington Hospital - Jefferson Health, Abington, USA

**Keywords:** gallbladder tumor, eus, endocrine tumor, chemotherapy

## Abstract

We report a case of rare and aggressive gallbladder neuroendocrine carcinoma (GB-NEC), diagnosed with the help of endoscopic ultrasound (EUS). A 65-year-old asymptomatic male, with a past medical history of hypertension, underwent abdominal ultrasound for the screening of an abdominal aortic aneurysm. He was found to have a mixed echogenicity area near the stomach, an incidental finding on abdominal ultrasound. The patient had an upper gastrointestinal (GI) endoscopy exam, which revealed an antral mass that was biopsied. The tissue specimen showed an epithelioid mesenchymal tumor of unclassified type and, eventually, the patient underwent partial gastrectomy. Surgical pathology reported a low-grade sub-serosal gastrointestinal stromal tumor (GIST) of the resected tissue specimen. He was later discharged and advised to follow up with abdominal computed tomography (CT) every year. Two years later, his abdominal CT revealed a new 3.7 cm x 2.0 cm mass in the posterior gallbladder fundus. Subsequently, the patient underwent laparoscopic cholecystectomy and the excisional biopsy reported a T3NXM1 neuroendocrine small cell carcinoma. Then, he received six cycles of systemic chemotherapy with carboplatin and etoposide, showing excellent response initially. However, a repeat CT abdomen/pelvis with contrast, on his eighth-month follow-up, demonstrated the interval development of an infiltrative mass in the pancreatic head. The gastroenterology team was then consulted, who performed sphincterotomy with temporary stent placement and celiac plexus neurolysis. Also, a transduodenal fine-needle aspiration (FNA) of the pancreatic mass was performed, which revealed metastatic small cell carcinoma. Based on these findings, the patient received an additional three cycles of carboplatin/etoposide chemotherapy, along with one cycle of immunotherapy. However, the patient had a poor response to chemotherapy, and he eventually chose hospice care.

## Introduction

Neuroendocrine tumors (NETs) comprise a group of rare, heterogeneous malignancies that present with varying histories and biological behaviors. Most of the times, these tumors emanate from the neuroendocrine cells present in the gastrointestinal (GI) and respiratory tracts but may originate from anywhere in the body. The neuroendocrine cells usually produce neuromodulators, neurotransmitters, and neuropeptide hormones.

Small cell carcinoma is a distinct but very unusual neoplasm of the gallbladder. The prevalence of gallbladder NEC is 1% to 5% of all gastrointestinal carcinoids. Here, we report a case of a neuroendocrine tumor of the gallbladder diagnosed by endoscopic ultrasound (EUS).

## Case presentation

**Figure 1:**
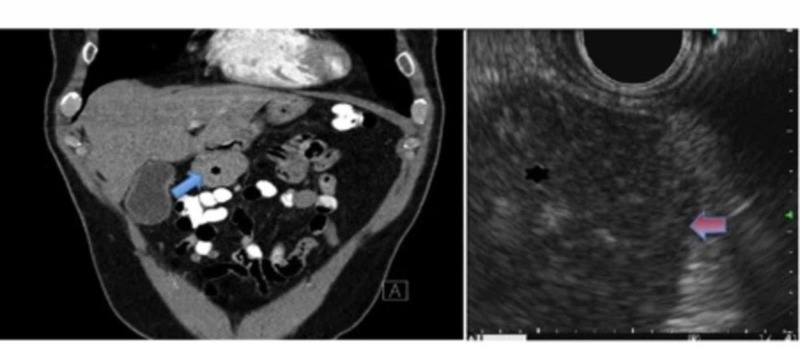
CT abdomen (left image) and EUS (right image) demonstrating GIST EUS: Endoscopic Ultrasound, CT: Computed Tomography, GIST: Gastrointestinal Stromal Tumor

A 65-year-old asymptomatic male, with a past medical history of hypertension, hyperlipidemia, and anxiety, presented to the primary care physician, for the screening of abdominal aortic aneurysm. Incidentally, the patient’s abdominal ultrasound revealed a mixed echogenic area near the stomach. It was later confirmed to be a 4.5-cm solid mass (Figure 1) between the stomach and the pancreas through an abdominal CT. An upper EUS revealed a 40-mm hypoechoic mass (Figure 1) on the lesser curvature of the stomach, with no sign of significant pathology in the main pancreatic duct and the pancreas.

The immediate onsite evaluation suspected a neuroendocrine tumor vs. a lymphoma, however, the transduodenal fine-needle aspiration (FNA) pathology reported an epithelioid mesenchymal tumor of unclassified type, negative for CD117 and DOG1, markers of GIST and other tumor types. Consequently, the GI tumor board recommended surgical resection, given the ambiguous biopsy results, unclear tissue origin, and the patient’s great performance status and lack of comorbidities. The patient underwent partial gastrectomy and the surgical pathology reported a low-grade sub-serosal gastrointestinal stromal tumor, with clear gastric muscularis, submucosal, and mucosal margins of resection. The tumor was positive for DOG1 and desmin and weakly positive for CD117 and smooth muscle actin. MIB1 showed approximately 1%-2% of the cells with nuclear immunoreactivity, indicating a low proliferative rate. Of note, the following markers were negative: AE1, CAM5.2, PAX8, CD34, S100, MelanA, HMB45, inhibin, EMA, myogenin, synaptophysin, and chromogranin. Postsurgical adjuvant chemotherapy was not indicated due to the low risk of recurrence, based on the modified National Institutes of Health (NIH) risk stratification criteria. The patient was then advised to follow up with an abdominal CT every year.

**Figure 2:**
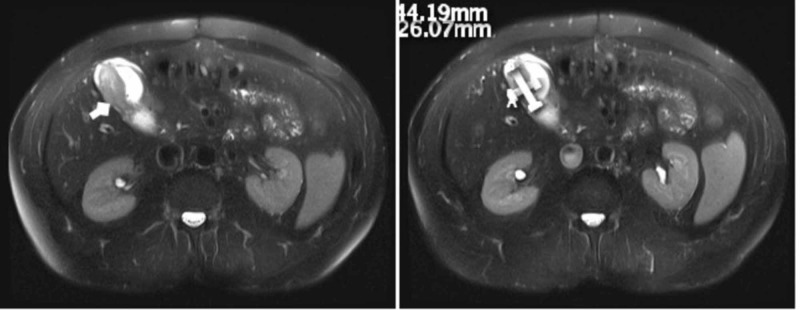
Posterior gallbladder fundus mass (white arrow) on MRI, initially suspected for GIST recurrence versus new malignancy. MRI: Magnetic Resonance Imaging, GIST: Gastrointestinal Stromal Tumor

Despite an initially negative surveillance one year later, a repeat CT after two years revealed a new 3.7 cm x 2.0 cm mass in the posterior gallbladder fundus, an 11-mm perihepatic node, and a 3.7-mm low-density lesion in the right hepatic lobe. Meanwhile, the patient developed new-onset nocturnal abdominal pain. Concerned for a possible GIST recurrence versus a new malignancy, an abdominal MRI was performed, which revealed a soft tissue mass along the gallbladder fundus/body, enlarging the pathologic portal caval, peripancreatic, and pericholecystic lymph nodes (Figure 2), consistent with metastatic disease.

A tumor markers’ workup revealed CEA 1.6 ng/ml, CA199 < 0.6, and chromogranin A 41 ng/ml, all within the normal range. To obtain a tissue sample, a repeat upper EUS was performed, which demonstrated one abnormal periportal lymph node, an irregular gallbladder mass, and a clear pancreas and common bile duct. FNA of the lymph node and the gallbladder mass demonstrated a small cell malignant neoplasm with a high nucleus-to-cytoplasm (NC) ratio and coarse nuclear chromatin. Unfortunately, extensive necrosis prevented the determination of a definite diagnosis. Since imaging studies were suggestive of aggressive pathology, the patient agreed to proceed with a laparoscopic cholecystectomy and wedge resection of a segment four liver lesion, in order to obtain an excisional biopsy. Pathology reported a T3NXM1 neuroendocrine small cell carcinoma, strongly positive for synaptophysin and CD56, with clear resection margins.

**Figure 3:**
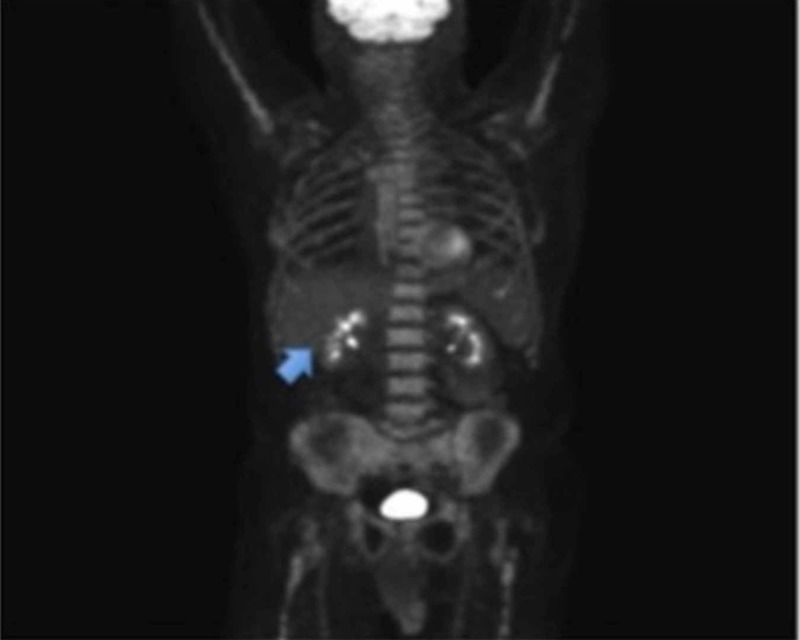
Initial PET scan following the resection of the gallbladder neuroendocrine tumor showing no residual mass or metastasis in pancreas and liver (arrow). PET: Positron Emission Tomography

Based on this information, the oncologist started the patient on systemic chemotherapy utilizing carboplatin and etoposide. The patient finished six cycles of chemotherapy with an excellent response. A follow-up PET/CT scan at three months and six months (Figure 3) revealed no fluorodeoxyglucose (FDG)-avid malignancy.

**Figure 4:**
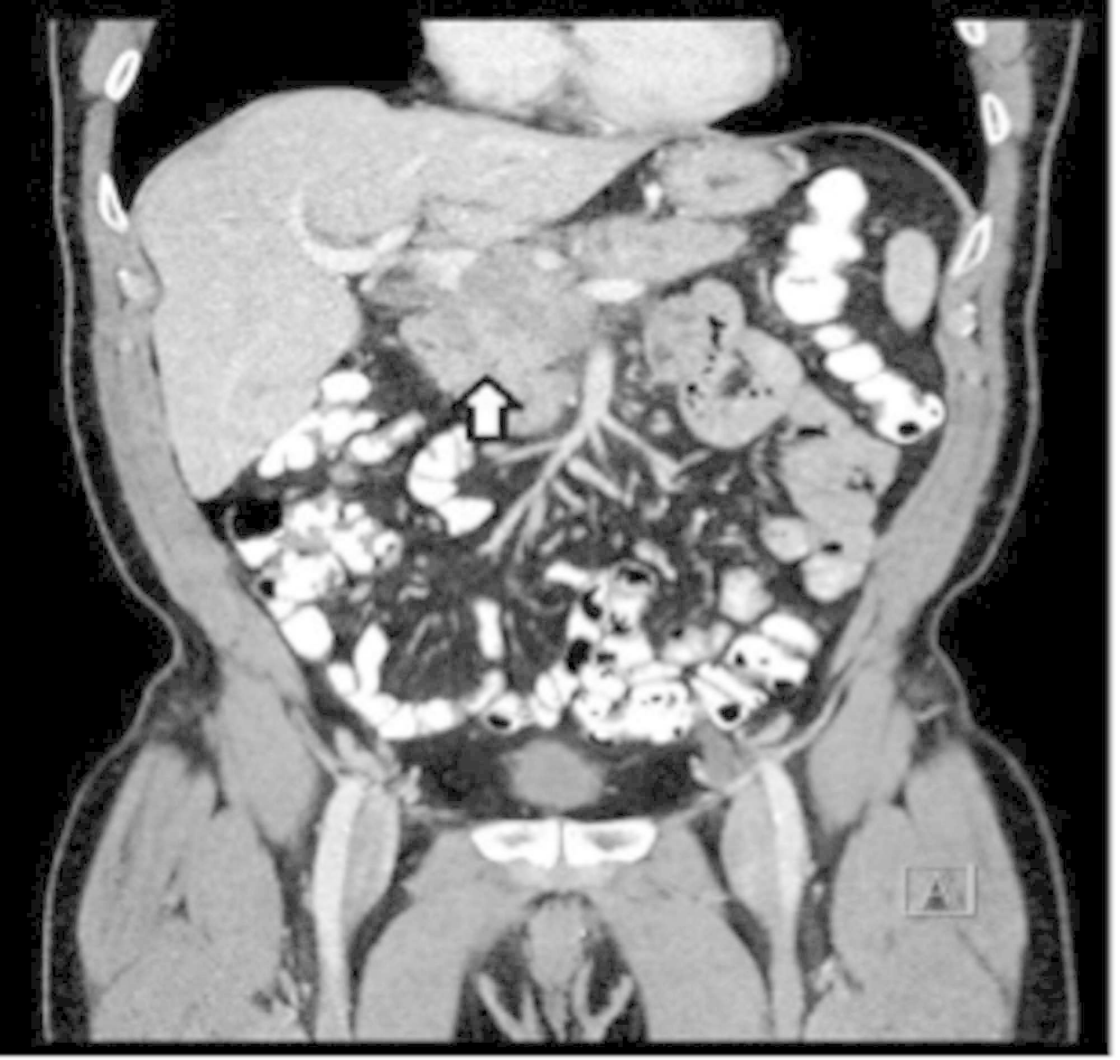
CT abdomen/pelvis showing interval development of an infiltrative pancreatic head mass (white arrow) s/p six cycles of chemotherapy. CT: Computed Tomography, s/p: Status Post

However, a repeat CT abdomen/pelvis, with contrast, at the eight-month follow-up demonstrated interval development of an infiltrative mass in the pancreatic head, peripancreatic lymphadenopathy, a hypodense lesion in the pancreatic tail (Figure 4), and new metastatic periportal, peripancreatic, and mesenteric adenopathy, concerning for metastasis. This was associated with obstructive intrahepatic biliary ductal dilatation and splenomegaly.

**Figure 5:**
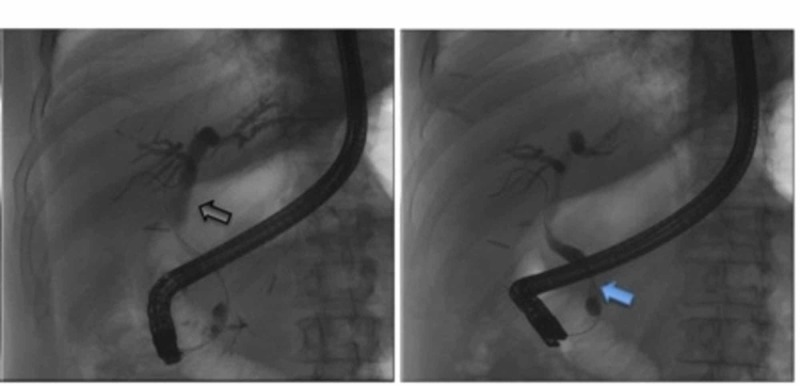
ERCP demonstrating distal narrowing of common bile duct (black arrow) and proximal dilatation (blue arrow) due to metastatic pancreatic head mass. ERCP - Endoscopic Retrograde Cholangiopancreatography

In addition, nine months postoperatively, the patient was admitted via the emergency department for acute abdominal pain, secondary to metastatic biliary obstruction. Blood work revealed an acutely elevated liver enzyme panel consistent with cholestasis. A EUS/endoscopic retrograde cholangiopancreatography (ERCP) (Figure 5) was performed during this hospitalization, which demonstrated a localized biliary stricture with malignancy.

Sphincterotomy and celiac plexus neurolysis were performed, and a temporary stent was placed. Pancreatic mass transduodenal FNA revealed a metastatic small cell carcinoma.

**Figure 6:**
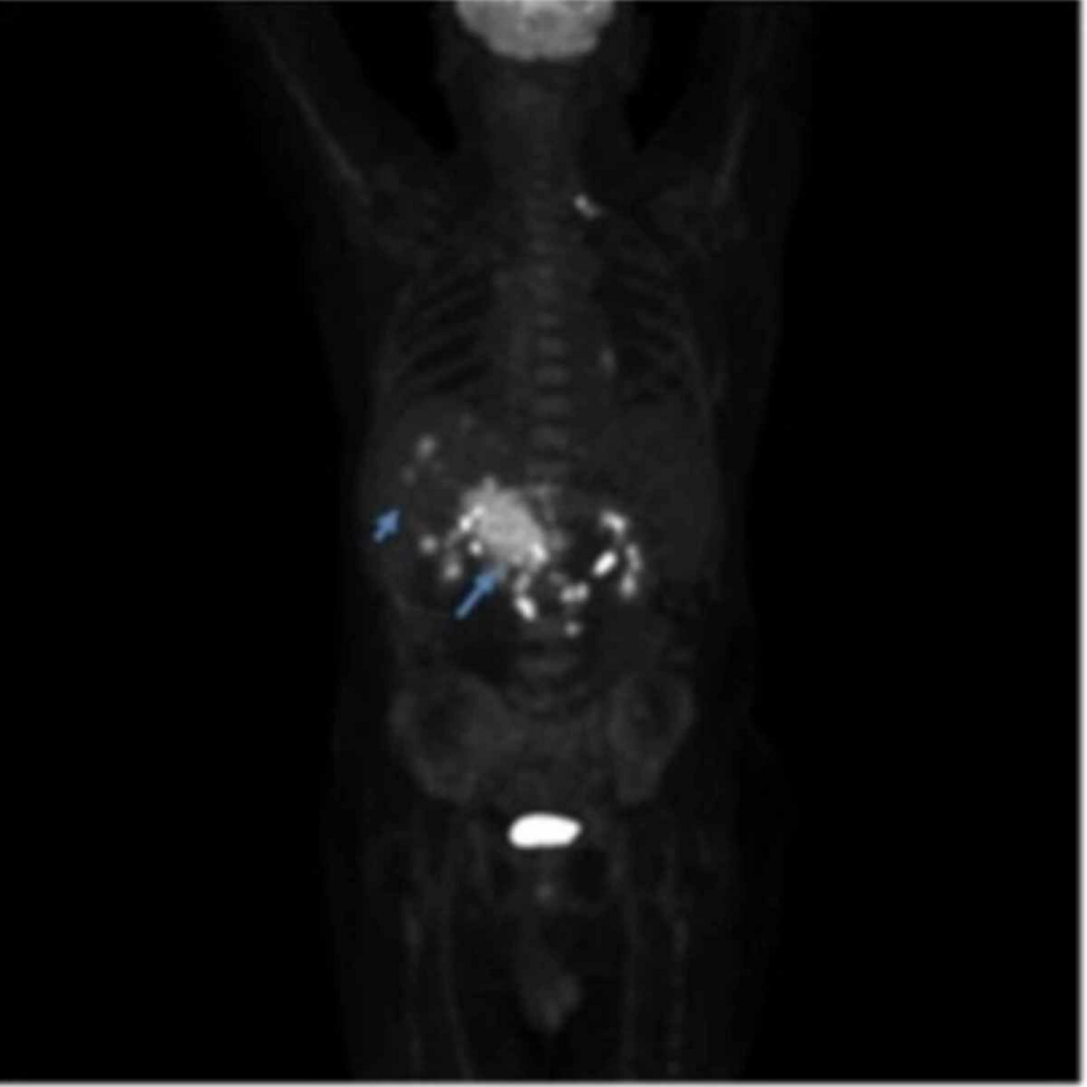
PET scan demonstrating metastatic GB-NEC (arrows) to liver and pancreas. GB-NEC: Gall Bladder Neuroendocrine Carcinoma, PET: Positron Emission Tomography

Despite aggressive management, the patient manifested with worsening epigastric pain and obstructive jaundice. He finished an additional three cycles of carboplatin/etoposide chemotherapy, together with one cycle of immunotherapy but showed a poor response, as evidenced by abdominal CT and PET/CT (Figure 6). Eventually, the patient elected for hospice care.

## Discussion

Neuroendocrine small cell carcinomas are very unusual endocrine tumors arising in the gastrointestinal tract and comprising less than 2% of all the primary gastrointestinal tumors [1]. Primary neuroendocrine tumors (NETs) frequently originate in the appendix, jejunum, and rectum, and less frequently involve the bronchial epithelium, duodenum, colon, and stomach. The involvement of the gall bladder is very infrequent and, in fact, rare. As per the Surveillance, Epidemiology, and End Results (SEER) program, neuroendocrine tumors of the gall bladder contribute to 0.5% of all NETs and only 2.1% of all gallbladder tumors [2]. A separate study by Duffy et al. included 435 patients who were diagnosed with gallbladder cancer between 1995 and 2005 and were treated at the Memorial Sloan-Kettering Cancer Center (MSKCC). The study showed 3% of these patients had NET [3].

Gallbladder neuroendocrine carcinomas (GB-NECs) have a higher incidence in women with age at presentation ranging from 35 to 80 years. According to most researchers, older females are at high risk of developing GB-NEC. A study by Ahn et al. revealed that only a few functional GB-NECs will have specific presentations [4]. Moreover, according to previous studies, most of them were non-functional, and the clinical presentation and signs of GB-NEC were non-specific [5-6]. Our patient had a non-secretory tumor, like most other small cell carcinomas, with no overt clinical signs related to secreted neuropeptides or any specific characteristics on the radiological studies. Matsuo et al. suggested that both pathology and immunohistochemical activity are necessary for a definite diagnosis [5]. Our patient was diagnosed with small cell carcinoma by both methods. A literature review of related case reports revealed 78% being pure small cell carcinoma and 22% small cell carcinoma combined with adenocarcinoma. According to SEER, the review of the pathological results of 41 GB-NETs (years 1973- 2004) indicated that the majority of the tumors (89.7%) were poorly differentiated or undifferentiated [6], only 2.4% were well-differentiated, and 7.3% were moderately differentiated.

Radiological imaging, such as CT, MRI, ultrasound (US), and tumor biomarkers, may not help in differentiating GB-NECs from other gallbladder tumors. Therefore, a biopsy should be obtained for immunohistochemical and pathological examinations. Currently, chromogranin A (CgA), synaptophysin (Syn), and neuro-specific enolase (NSE) are the most commonly used immunological biomarkers for NECs. Neuroendocrine carcinomas are very malignant tumors that progress rapidly, resulting in early liver invasion and lymphatic metastasis. Most GB-NETs are identified incidentally during a routine histological examination of gall bladder samples after surgical excision and are often accompanied by cholelithiasis and cholecystitis. Literature suggests that the origin of NETs is supported by the presence of metaplasia in the gall bladder due to chronic irritation, caused by cholecystitis. This hypothesis is supported by the histology and epithelial origin of the tumor. Since the normal gallbladder does not typically contain neuroendocrine cells, these tumors are very rarely found.

The gold standard treatment for GB-NET involves surgery for the total removal of the gallbladder. Various surgical procedures can be used, ranging from simple cholecystectomy to extensive radical resection (including local lymph node removal and resection of metastases) [2]. While simple cholecystectomy is mandated for pre-invasive and early-detected cancers (T1s and T1), a more aggressive surgery in the form of radical cholecystectomy and regional lymphadenectomy, combined with a hepatic resection with adequate free margins, is always performed for advanced lesions [7].

In a study by MSKCC researchers, involving 13 patients with GB-NEC, the median patient survival time was 9.8 months, which was not significantly different from the median survival time of patients (n=435) with gallbladder carcinomas (10.3 months) [8]. A study by Fujii et al. showed a poor survival rate of 28% and 0% at the end of one and two years, respectively, among 53 patients with small cell gallbladder cancers. These findings suggest a poorer prognosis for GB-NEC as compared to gallbladder adenocarcinoma, possibly due to a greater percentage of patients at an advanced disease stage and with lymphatic metastases at the time of diagnosis.

Literature regarding the prognosis of GB-NEC is relatively limited. Some studies revealed that elevated Ki-67, a high mitotic index, and invasion into adjacent structures predict poor outcome and prognosis. Despite major surgical removal, a majority of the patients can still develop recurrent metastatic disease [9-10]. Moskal et al. [11] documented that the most common metastatic sites for GB-NET are lymph nodes (88%), liver (88%), lung (23%), and peritoneum (19%). In a previous study on 12 patients who underwent cholecystectomy, median survival was 4.5 months. A radical resection performed on two GB-NEC-SCC (small cell neuroendocrine carcinoma of the gall bladder) patients resulted in survival periods of four and 20 months [3]. According to previous research, a significant advantage in the five-year survival rate was observed in patients with the T2 stage undergoing the radical operation as compared to those who received only simple cholecystectomy (38%-100% vs 17%-65%) [12].

Most patients with GB-NEC are diagnosed at late stages, as most NETs are highly invasive and have a high risk of lymphatic metastasis, which decreases the rate of radical resection. Platinum-based chemotherapy is the first-line chemotherapeutic agent. A study by Elahi et al. [13] reported that the survival time with highly differentiated GB-NEC was 46 months when postoperative chemotherapy was combined with the application of cisplatin, docetaxel, gemcitabine, and sunitinib. Another study by Okuyama et al. [14] described a patient survival time of 22 months after the combined application of cis-platinum and docetaxel. These findings suggest that adjuvant radiotherapy and chemotherapy may benefit patients with GB-NEC. Few cases of GB-NEC have been reported universally; therefore, no preferred chemotherapy and radiotherapy strategies have been suggested.

New targeted therapies are being developed for NETs since traditional chemotherapy became ineffective. Recent advancements include the involvement of tyrosine kinase inhibitors in advanced gastro-entero-pancreatic tumors [15-17]. NETs express several growth factor receptors, including estimated glomerular filtration rate (EGRF), RAC-a serine/ threonine-protein kinase (AKT), stem cell factor receptor c-Kit, mTOR, and insulin-like growth factor (IGF), which can be downregulated by tyrosine kinases. Various targeted therapies, including TK receptor inhibitors (imatinib, gefitinib), mTOR blockers (temsirolimus, everolimus), and those targeting angiogenesis (sorafenib, sunitinib, vatalanib), are being investigated for gastro-entero-pancreatic neuroendocrine tumors [18].

## Conclusions

In summary, GB-NECs are rare gallbladder tumors, with a low rate of incidence and a non-specific clinical presentation. Therefore, pathological and immunohistochemical examinations are needed to make a definite diagnosis. GB-NECs are highly malignant tumors and can present with lymphatic metastases and local invasion during the early stages. The prognosis of GB-NEC is usually poor as compared to a gallbladder adenocarcinoma. Therapy with a combination of surgical resection, radiotherapy, and chemotherapy can potentially increase patient survival. However, no universally accepted guidelines of treatment are available because of its low incidence and few relevant studies. Further studies with a larger sample size are needed to explore efficient treatment strategies.
